# Impact of Domestication on Floral Traits and Rewards: A Comparison Between Wild and Domesticated Squash (*Cucurbita*)

**DOI:** 10.1002/ece3.72028

**Published:** 2025-08-29

**Authors:** Luis Alberto Villanueva‐Espino, Irais Avila‐Eulogio, M. Hesajim de Santiago‐Hernández, César Mateo Flores‐Ortiz, Rafael Lira Saade, Adonaji Cortés Pérez, Eric J. Fuchs, Mauricio Quesada

**Affiliations:** ^1^ Laboratorio Nacional de Análisis y Síntesis Ecológica, Escuela Nacional de Estudios Superiores Universidad Nacional Autónoma de México (UNAM) Morelia Michoacán Mexico; ^2^ Posgrado en Ciencias Biológicas, Unidad de Posgrado, Edificio D, 1° Piso, Circuito de Posgrados Ciudad Universitaria Ciudad de México Mexico; ^3^ Laboratorio Binacional de Análisis y Síntesis Ecológica Universidad Nacional Autónoma de México‐Universidad de Costa Rica (UNAM‐UCR) Morelia Michoacán Mexico; ^4^ Instituto de Investigaciones en Ecosistemas y Sustentabilidad Universidad Nacional Autónoma de México Morelia Michoacán Mexico; ^5^ Laboratorio de Vida Silvestre, Facultad de Biología Universidad Michoacana de San Nicolás de Hidalgo Morelia Michoacán Mexico; ^6^ Unidad de Biotecnología y Prototipos (UBIPRO), Facultad de Estudios Superiores Iztacala Universidad Nacional Autónoma de México Tlalnepantla Estado de México Mexico; ^7^ Escuela de Biología Universidad de Costa Rica San José San Pedro de Montes de Oca Costa Rica

**Keywords:** artificial selection, flower morphology, nectar, phenotypic variation, pollen

## Abstract

Plant domestication primarily targets traits of direct human interest, such as fruit and seed characteristics; however, its indirect effects on other traits, including floral morphology and rewards (nectar and pollen), remain less understood. In this study, we investigated how domestication has influenced floral traits and rewards in domesticated and wild species of the genus *Cucurbita*. We compared three domesticated and three wild *Cucurbita* species in an experimental plot. We measured floral morphological traits, nectar volume, sugar (fructose, glucose, and sucrose), and amino acid concentrations in staminate and pistillate flowers. In addition, we evaluated pollen production and size, as well as protein and lipid concentrations, and the protein: lipid ratio in staminate *Cucurbita* flowers. Our results show that domesticated *Cucurbita* species exhibit larger floral morphological traits in both pistillate and staminate flowers compared to their wild relatives. While nectar volume increased in domesticated species, sugar and amino acid concentrations remained unchanged. In contrast, domestication had no significant effect on pollen traits, including production, size, and protein and lipid content. These findings highlight that domestication differentially affects floral traits: while floral morphology is significantly altered, most of the traits of floral rewards remain largely unaffected. This conservation may arise from the recent evolutionary history of these species and their close coevolutionary relationship with *Eucera* bees, emphasizing pollinator nutritional needs over artificial selection. These results underscore the complex interplay between domestication, resource allocation, and plant–pollinator interactions in shaping floral traits.

## Graphical abstract

Plant domestication primarily targets traits of direct human interest, but also indirectly affects other traits. We investigated the effect of domestication on floral traits and floral rewards of species of the genus *Cucurbita*. Multivariate Principal Component Analysis shows that floral morphological traits differ between domesticated and wild species (A‐pistillate and B‐staminate flowers) and between species (C‐pistillate and D‐staminate flowers). However, nectar and pollen traits remain largely unaffected. Therefore, domestication is affecting floral morphological traits but not reward traits.

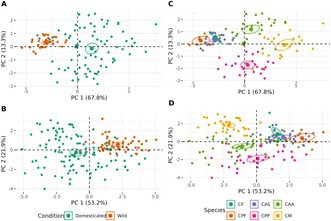

## 1 Introduction

Plant domestication is an evolutionary process in which humans select desirable plant phenotypes or characteristics for food or utility purposes. This artificial selection is influenced by integrated ecological, biological, and cultural factors (Darwin 1859; Zeder 2006; Casas et al. 2007; Brown 2010; Meyer et al. 2012; Larson et al. 2014; Purugganan 2022). The complex of traits that differentiate domesticated plants from their wild relatives is called “domestication syndrome” (Fuller 2007; Pickersgill 2007; Meyer et al. 2012; Larson et al. 2014). These traits mainly include increased fruit and seed size, loss of seed dispersal and dormancy, and reduction of physical and chemical defense (Pickersgill 2007; Brown 2010; Larson et al. 2014).

Selection for traits beneficial to humans may indirectly impact the expression of other nontarget traits (Milla et al. 2015; Prieto et al. 2017; Hernández‐Terán et al. 2017; Serrano‐Mejía et al. 2022). These changes in nontarget traits could be the result of different factors, including pleiotropic effects (Filipecki and Malepszy 2006), developmental constraints (Pérez‐Barrales et al. 2007), or metabolic regulations (Hernández‐Terán et al. 2017). These nontarget traits may influence fitness and therefore hinder the evolution of domesticated plants (Milla et al. 2015; Serrano‐Mejía et al. 2022). However, there is little information on the effect of domestication on nontarget traits, such as those related to reproductive function (e.g., floral traits and rewards; Kuriakose et al. 2009; Solís‐Montero et al. 2021; Glasser et al. 2023). For example, in cardamom ( *Elettaria cardamomum* L.), human‐induced selection in cultivated plants targeted an increase in fruit size, indirectly increasing flower length, labellum size, corolla tube, nectar quantity, and sugar concentration when compared to the same traits of wild plants of the same species (Kuriakose et al. 2009). In *Physalis philadelphica* (husk tomato), the increase in fruit size in domesticated plants also results in an increase in flower size and more production of ovules than in semi‐domesticated or wild plants (Solís‐Montero et al. 2021). Likewise, Glasser et al. (2023) determined that flowers of two domesticated species of *Cucurbita* were larger and had higher correlations among traits than those of their wild relatives. Understanding the impacts of domestication on floral traits and floral rewards will help us understand how plants respond to artificial selection. Additionally, it will help us develop breeding strategies that balance desirable traits with ecological interactions and plant fitness.

The primary floral rewards offered by plants to pollinators are nectar and pollen. Nectar is the most common floral reward and is considered a mediator of plant–pollinator interactions (Carter and Thornburg 2004; Kaczorowski et al. 2005; Herrera et al. 2006; Nepi 2017; Roy et al. 2017; Chatt et al. 2018; Parachnowitsch et al. 2019; Nicolson 2022). Nectar is mainly composed of sugars (sucrose, glucose, and fructose) and amino acids, in addition to secondary metabolites, such as alkaloids, phenols, terpenes, and flavonoids (Carter and Thornburg 2004; Nepi et al. 2018; Palmer‐Young et al. 2019). The other floral reward of great importance is pollen, whose primary function is to transport the male gametes of the plant (Nepi et al. 2018; Treanore et al. 2019; Ruedenauer et al. 2019; Rivest and Forrest 2020); however, it is also exploited by pollinators, mainly as a nutritional resource for adult and larval development, as it contains proteins, lipids, amino acids, carbohydrates, and vitamins (Vanderplanck et al. 2014; Somme et al. 2015; Venjakob et al. 2022). To date, few studies have assessed whether domestication processes may affect these floral rewards. For example, in blueberries ( *Vaccinium corymbosum* L.), domestication significantly altered the chemical composition of nectar and pollen, reducing the chemical diversity of pollen in cultivated plants (Egan et al. 2018). Domestication‐induced changes in the chemistry of floral rewards may affect pollinator physiology, ecology, and behavior (Palmer‐Young et al. 2019).

The genus *Cucurbita* L. is native to the Americas (Nee 1990; Gong et al. 2012), comprising 20 taxa belonging to 15 species (Lira‐Saade 1995; Lira et al. 2016). Five of these species were domesticated through six independent domestication events (Sanjur et al. 2002; Smith 2006; Kistler et al. 2015; Kates et al. 2017; Castellanos‐Morales et al. 2018), with Mexico being the center of origin and diversification (Lira et al. 2016; Kates et al. 2017; Sánchez‐de La Vega et al. 2018; Martínez‐González et al. 2021). These cucurbit species are now distributed worldwide and represent one of humans' most important food sources (Balvino‐Olvera et al. 2017). The genus *Cucurbita* is divided into two ecological groups: perennial xerophytic species that have storage roots and are basal in the phylogeny, and annual mesophytic species having no storage roots that include all domesticated species (Nee 1990; Zheng et al. 2013; Lira et al. 2016; Aguirre‐Dugua et al. 2023). *Cucurbita* species are predominantly monoecious, except for *C. foetidissima* , which is gynodioecious (Kohn and Biardi 1995), and depend on pollinators, mainly *Eucera* bee species (Dorchin et al. 2018; Delgado‐Carrillo et al. 2018; Freitas et al. 2023). The flowers are singly in leaf axils, gamopetalous with bell‐shaped corollas, usually with orange to yellow coloration (Paris 2016). Staminate flowers exhibit fused anthers in a filament column, while pistillate flowers exhibit an inferior ovary, and the pistillate complex has three fused bilobed styles with open nectaries. Staminate flowers have long, slender pedicels, and pistillate flowers have short, thick pedicels (Lira‐Saade 1995; Lira et al. 2016). Flowers open at dawn and fade at noon (Paris et al. 2012). *Cucurbita* cultivars differ primarily from their wild relatives by having less branched growth, larger fruits and seeds, thicker, less fibrous pulp, and the absence of cucurbitacins (Gong et al. 2012; Paris et al. 2012; Lira et al. 2016; Paris 2016). Domesticated species may also differ in other traits, as they have been selected for different uses (Jaccard et al. 2021). Some species are cultivated for immature fruits, while others are grown for their ripe pulp and seeds. Additionally, some species are cultivated solely for their seeds (Gong et al. 2012; Paris 2016). The genus *Cucurbita* is an ideal model for studying plant domestication and its indirect effects on reproductive traits, including potential impacts on pollinators, reproductive success, and fitness, because it has phylogenetically related domesticated and wild species growing in sympatry in its range of distribution (Lira et al. 2016).

The main objective of this study was to examine the impact of the domestication process on the floral morphological traits of both pistillate and staminate flowers, as well as on floral rewards, including nectar and pollen. This was accomplished by comparing three domesticated species with three phylogenetically related wild species. We compared floral morphological traits between domesticated and wild species of pistillate and staminate flowers. Artificial selection in the genus *Cucurbita* has mainly targeted fruit and seed traits; concomitantly, we expect that indirect selection will have a greater effect on floral traits in domesticated than in wild species. Regarding the floral reward, and considering the critical role of nectar and pollen chemical composition in plant fitness, we expect a weak effect of artificial selection on these traits between wild and domesticated *Cucurbita* species.

## 2 Materials and Methods

### 2.1 Study Species

We selected six species of the *Cucurbita* genus, three domesticated species and three wild species. Within the Pepo clade, we selected the domesticated species *C. pepo* subsp. *pepo* (CPP) and its wild relative *C. pepo* subsp. *fraterna* L. H. Bailey (CPF). From the Argyrosperma clade, we selected two domesticated species, *C. argyrosperma* subsp. *argyrosperma* Huber (CAA) and *C. moschata* Duchesne (CM), and their wild relative within the Argyrosperma clade, *C. argyrosperma* subsp. *sororia* L. H. Bailey (CAS). We selected the xerophytic species *C. foetidissima* Kunth (CF), which belongs to the basal clade Foetidissima (Figure S1).

### 2.2 Plant Material and Experimental Design

Seeds of the wild species (CF, CPF, and CAS) were sampled from wild populations in the states of Guanajuato, Tamaulipas, and Jalisco. The domesticated species CAA and CM were obtained from local farmers in the states of Jalisco, Michoacán, and Guerrero. Finally, we obtained 99% pure CPP seeds from a commercial brand (EDENA seeds, El Centro, CA, USA).

The study was conducted during the wet season of 2021 to 2023 in Morelia, Michoacán, Mexico (19.652174 N, −101.224311 W). Seeds of the six species were germinated in a greenhouse at the Laboratorio Nacional de Análisis y Síntesis Ecológica (LANASE), Universidad Nacional Autónoma de México, campus Morelia (Michoacán, México), under controlled temperature and humidity conditions. Three weeks after germination, 20 seedlings of each species with at least two developed leaves were transplanted to an experimental field in a common garden experiment. Each seedling was planted at a 3 m distance between rows and 3 m between plants within the same row. The experiment was established as a randomized complete block design with 20 replications. Each replicate consisted of one plant of each species sown randomly (*N* = 120; Figure S2).

### 2.3 Floral Morphological Traits

**FIGURE 1:**
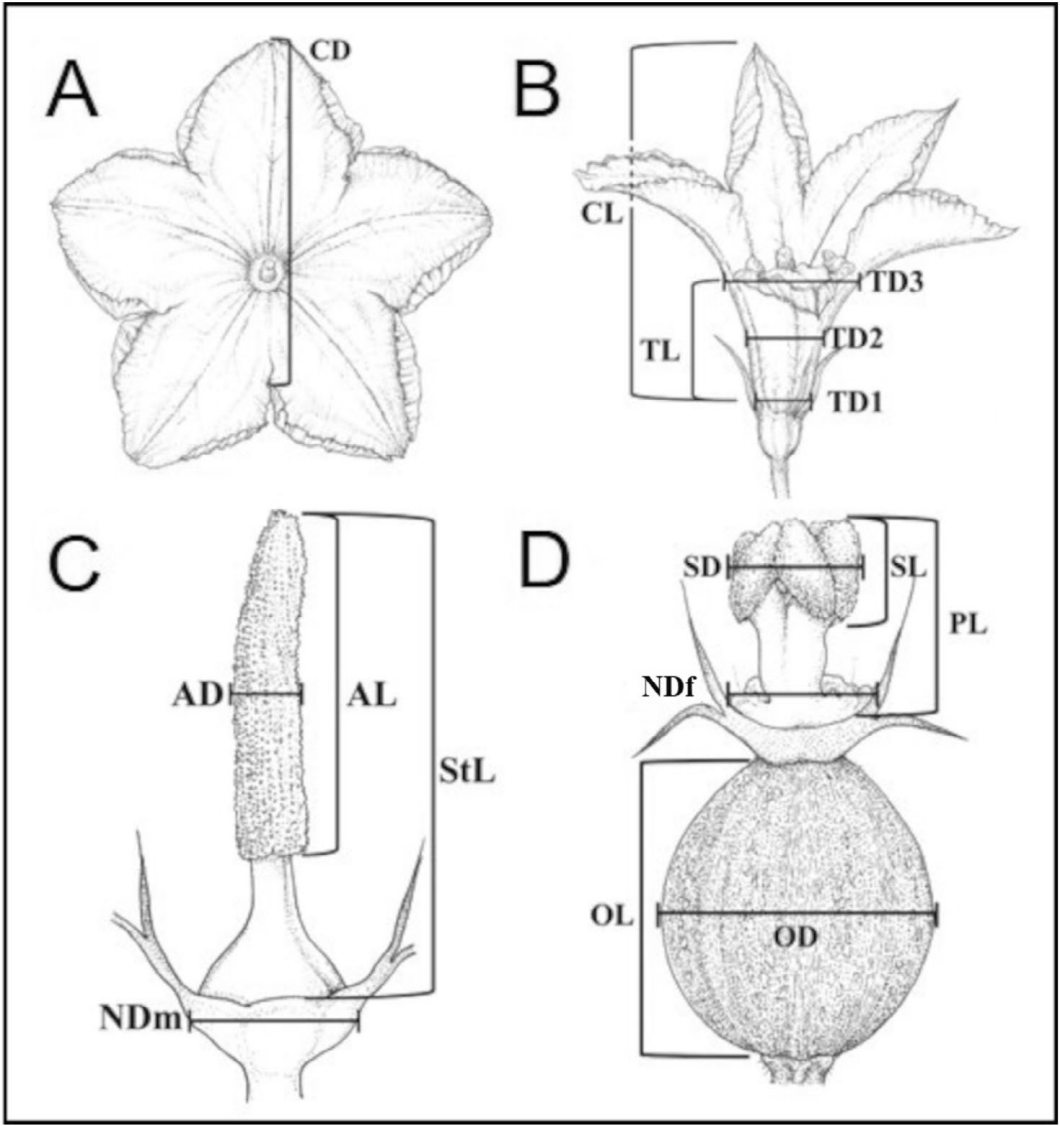
Sketch of morphological traits measured in pistillate and staminate *Cucurbita* flowers, taken from Glasser et al. (2023). A) Corolla diameter (CD) of *Cucurbita* flowers, B) lateral view of a staminate flower, C) traits that form the androecium of staminate flowers, and D) traits that form the gynoecium of pistillate flowers.

To compare flower morphological traits between domesticated and wild species of *Cucurbita*, we measured 16 morphological floral traits as suggested by Glasser et al. (2023). Before anthesis, flower buds were covered with a mesh bag to prevent insect visitation. Floral traits were measured in fully open flowers from 7:00 to 12:00 h. All measurements were made with a digital vernier caliper (0.1 mm accuracy, HER‐411, Steren, China). In pistillate flowers, we measured corolla diameter (CD), corolla tube length (TL), corolla length (CL), corolla tube base diameter (TD1), corolla tube middle diameter (TD2), corolla tube end diameter (TD3), nectary diameter (NDf), stigma diameter (SD), stigma length (SL), pistil length (PL), ovary length (OL), and ovary diameter (OD; Figure 1). In staminate flowers, we measured corolla diameter (CD), corolla tube length (TL), corolla length (CL), corolla tube base diameter (TD1), corolla tube middle diameter (TD2), corolla tube end diameter (TD3), nectary diameter (NDm), anther diameter (AD), stamen length (StL), and anther length (AL; Figure 1).

### 2.4 Nectar Traits

To compare nectar traits between domesticated and wild *Cucurbita* species, we recorded the nectar volume of all measured flowers. Nectar was extracted from each flower using a 10 μL micropipette (Finnpipette F1, Thermo Scientific, Finland). To determine the sugar composition (fructose, glucose, and sucrose) and concentration in the floral nectar of *Cucurbita* species, we used the chemometric method described by Flores‐Ortiz et al. (2003).

We prepared 125 sugar‐standard solutions with varying concentrations of fructose, glucose, and sucrose, ranging from 0% to 20% (w/v). Five μL of each sugar‐standard solution were measured using a Frontier FT‐IR/NIR spectrometer (Perkin Elmer, Boston, MA, USA) equipped with an UATR accessory. We used an infrared spectrum ranging from 4000 to 650 cm^−1^ with a resolution of 4 cm^−1^. The spectrum obtained was the average of 32 spectra. To construct the chemometric model, the set of 125 standard sugar solutions was analyzed by multidimensional statistical analysis with Spectrum software (Perkin Elmer) using the Partial Least Squares algorithm. The spectra were subjected to normalization, baseline correction by the second derivative, and suppression of the water and carbon dioxide signals. Once the chemometric model was constructed, we estimated the concentrations of the different sugars in the nectar samples of each species (*n* = 83 nectar samples in total). In order to validate the total sugar quantification using the chemometric model, the total amount of sugars in the nectar samples was measured using a pocket refractometer PAL‐1 (Atago, Tokyo, Japan; Figure S3). Each sample measured with the refractometer was diluted 1:10 and measured at approximately 25°C.

To compare concentrations of amino acids in floral nectar between domesticated and wild *Cucurbita* species, we conducted high‐performance liquid chromatography (HPLC) to determine the concentration of 17 free amino acids in the floral nectar of the different *Cucurbita* species. The measured amino acids were: aspartic acid (Asp), glutamic acid (Glu), serine (Ser), histidine (His), glycine (Gly), threonine (Thr), arginine (Arg), alanine (Ala), tyrosine (Tyr) cysteine (Cys), valine (Val), methionine (Met), phenylalanine (Phe), isoleucine (Ile), leucine (Leu), lysine (Lys), and proline (Pro).

Analyses were performed on an Agilent 1100 Series (Hewlett Packard) following the Agilent 1090 series method; we used a two‐step precolumn derivatization with ortho‐phthalaldehyde (OPA) for primary amino acids and 9‐fluorenylmethyl chloroformate (FMOC) for the secondary amino acid (Rodríguez‐Peña et al. 2013, 2021). In brief, a 0.4 N borate buffer was used with a pH of 10.4. Each amino acid was separated using an InfinityLab Poroshell HPH‐C18 4.6 × 100 mm 2.7 μm column (Agilent).

A gradient with two mobile phases was used. The first mobile phase (A) consisted of a solution of sodium phosphate, sodium borate, and sodium nitride (pH 8.2), and the second mobile phase (B) consisted of a mixture of methanol, acetonitrile, and water in a ratio of 45:45:10. The gradient started with 98% A and 2% B; at 13.40 min, 43% A and 57% B; at 13.50 min, 0% A and 100% B; at 15.80 min, 98% A and 2% B with a constant flow rate of 1500 mL/min.

The detection was performed using an Agilent 1100 series Diode Array Detector (DAD, G1315A). Agilent OpenLAB CDS ChemStation (1.9.0) software collected the data. We ran pure amino acid standards with known concentrations and recorded their retention times and peak heights. We ran the samples and noted the peaks with exact retention times as those found on the standards. To calculate the concentration of amino acids, we divided the peak height of each amino acid in the samples by the peak height of each corresponding standard and multiplied it by the standard's concentration (μg/mL).

### 2.5 Pollen Traits

To compare pollen production and pollen size between species, we collected three flowers from 4 to 14 plants per species (*N* = 59 plants) due to differences in flower availability and production among species (Appendix S1). Flowers near anthesis were cut before the anthers dehisced to avoid pollen loss. The anthers were deposited in 1.5 mL tubes. Anthers were dried at an average temperature of 32°C for 1 week and stored in 1.5 mL tubes until analysis. Before pollen quantification, we rehydrated anthers with a 2% NaCl solution and centrifuged them at 13,000 rpm for 10 min or until all pollen was released from the anthers. Pollen grains from each flower were quantified and measured using an ELZONE II 5390 particle counter (Micromeritics, Norcross, GA, USA) using a tube with a 300 μm aperture. We measured the equatorial diameter of 100 pollen grains per *Cucurbita* species using an optical microscope (Axio Imager A2, Zeiss, New York, USA) to determine the mean and variation in pollen grain sizes. Each sample was quantified three times, and the total pollen production for each flower was calculated by averaging the three measurements.

To compare protein and lipid concentrations in pollen between domesticated and wild *Cucurbita* species, and given differences in flower availability and production for each species (Appendix S1), we collected three flowers from 8 to 15 plants per species (*N* = 63 plants). Anthers were stored in 2 mL tubes and kept at −20°C for subsequent analysis. One milligram of each pollen sample was used to perform both protein and lipid analysis. Protein concentration was analyzed using the Bradford assay, and lipid concentration was determined using the assay of Van Handel and Day (1988), as modified by Vaudo et al. (2016, 2024). Protein and lipid concentrations in pollen were reported as micrograms of nutrients per milligram. The protein:lipid ratio (P:L) was calculated by dividing protein concentration by lipid concentration (Vaudo et al. 2016, 2024).

### 2.6 Statistical Analysis

To identify patterns of morphological variation among the floral traits of domesticated and wild *Cucurbita* species in pistillate and staminate flowers, we conducted a Principal Component Analysis (PCA) with the *stats* package (R Core Team 2024). We then used a permutational multivariate analysis of variance (PERMANOVA), as implemented in the *vegan* R library (Oksanen et al. 2022), to compare floral traits between conditions (wild and domesticated) and among *Cucurbita* species.

We performed generalized linear models (GLM) to compare nectar volume, total sugar concentration, and the concentration of fructose, glucose, and sucrose between conditions and among species. The dependent variables were nectar volume, percentage of total sugar, and amounts of sucrose, glucose, and fructose, whereas the independent variables were species, floral sex, and their interactions. To identify the patterns of variation in the concentrations of all amino acids between domesticated and wild species, as well as floral sex, we performed a PCA with the concentration of each of the 17 amino acids. To determine differences between conditions and among species and floral sex in amino acid concentrations, we performed a PERMANOVA. We conducted GLMs to compare pollen production, size, protein concentration, lipid concentrations, and P:L ratio between conditions and among species.

To determine the effect of domestication on floral traits and to account for the phylogenetic effect on these traits, first, we reconstructed the phylogeny of our study species from the phylogeny of Castellanos‐Morales et al. (2018) using the phytools (Revell 2024) and jpeg (Urbanek 2022) packages. We tested for a phylogenetic signal with Pagel's λ and Blomberg's *K* using the phylosig command from the phytools package. These indexes measure the tendency of related species to resemble each other (Pagel 1999). However, the results from these analyses showed that floral traits do not have a significant phylogenetic signal (Tables S2 and S3). All statistical analyses were performed with R, version 4.4.2 (R Core Team 2024).

## 3 Results

### 3.1 Floral Morphological Traits

**FIGURE 2:**
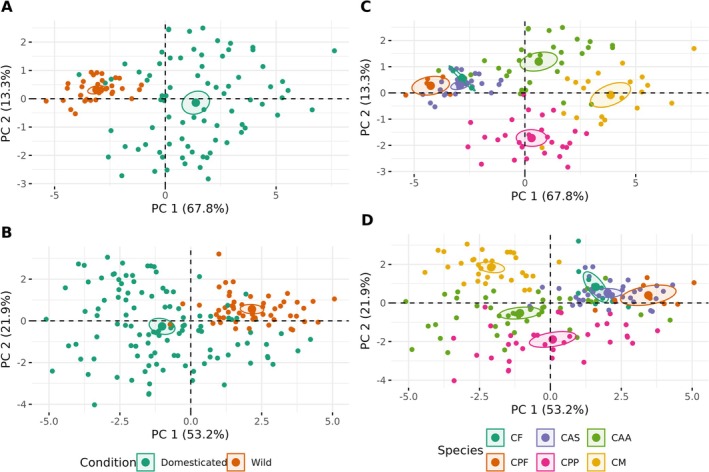
Principal component analysis of floral traits by conditions (wild & domesticated) and by species for pistillate (A, C) and for staminate flowers (B, D), respectively. The wild species are CF ( *C. foetidissima* ), CPF ( *C. pepo* subsp. *fraterna*), and CAS ( *C. argyrosperma* subsp. *sororia*). The domesticated species are CPP ( *C. pepo* subsp. *pepo*), CAA ( *C. argyrosperma* subsp. *argyrosperma*), and CM ( *C. moschata* ).

For floral morphology analyses, we measured 349 pistillate and 883 staminate flowers in 280 plants from six different species (Table 1). The results of the PERMANOVA analysis showed that pistillate flowers differed significantly between conditions (*F* = 68.05, df = 1, *p* < 0.001; Figure 2A) and among species (*F* = 32.684, df = 5, *p* < 0.001; Figure 2C), with domesticated species exhibiting larger flowers than wild species. The PC1 explained 67.8% of the variance, and the traits that contributed the most to this variation were stigma diameter (SD), stigma length (SL), and corolla tube middle diameter (TD2). The PC2 explained 13.3% of the total variance, and the traits that contributed the most to this component were ovary length (OL), corolla tube base diameter (TD1), and tube length (TL). We observed similar results in the staminate flower phenotypes. The PERMANOVA showed statistical differences between conditions (*F* = 79.16, df = 1, *p* < 0.001; Figure 2B) and among species (*F* = 35.736, df = 5, *p* < 0.001; Figure 2D), where domesticated species have larger flowers than wild species. The PC1 explained 53.2% of the total variance, and the traits that contributed the most to the variation were corolla length (CL), corolla diameter (CD), and corolla tube end diameter (TD3). While PC2 explained 21.9% of the variance, the traits that contributed the most were anther length (AL), nectary diameter (Ndm), and stamen length (StL).

**TABLE 1:** Mean (±SD) of morphological traits of pistillate and staminate flowers of domesticated and wild *Cucurbita* species: *C. foetidissima* (CF), *C. pepo* subsp. *fraterna* (CPF), *C. argyrosperma* subsp. *sororia* (CAS), *C. pepo* subsp. *pepo* (CPP), *C. argyrosperma* subsp. *argyrosperma* (CAA), and *C. moschata* (CM). N is the number of plants measured in each species. Different letters within a row indicate differences between species.

Floral sex	*Cucurbita* species					
Pistillate	Wild			Domesticated		
Floral traits	CF (*N* = 3)	CPF (*N* = 5)	CAS (*N* = 27)	CPP (*N* = 27)	CAA (*N* = 31)	CM (*N* = 21)
CD	49.60 ± 1.72a	53.78 ± 8.02a	69.97 ± 8.08a	85.64 ± 14.77b	98.65 ± 18.84c	104.03 ± 14.76c
TL	41.90 ± 6.56a	40.06 ± 4.56a	41.41 ± 4.30a	43 ± 6.53a	55.91 ± 8.18b	62.39 ± 9.45b
CL	65.95 ± 3.76ab	57.35 ± 4.92a	70.99 ± 7.04b	74.08 ± 11.34b	88.29 ± 12.01c	100.83 ± 16.24d
TD1	17.15 ± 1.68ab	14.39 ± 1.34a	16.45 ± 1.80a	26.21 ± 3.98c	19.83 ± 4.85b	28.89 ± 4.02c
TD2	24.77 ± 1.97ab	22.99 ± 3.48a	24.15 ± 3.61a	37.66 ± 4.77cd	34.34 ± 8.43bc	43.10 ± 4.94d
TD3	29.13 ± 2.29a	34.13 ± 6.13a	39.37 ± 6.26a	52.77 ± 8.83b	62.36 ± 12.28c	65 ± 12.37c
NDf	9.76 ± 0.72ab	9.09 ± 1.27a	11.77 ± 1.35b	18.53 ± 2.50d	14.06 ± 2.38c	21.68 ± 2.74e
SD	14.31 ± 2.75abc	11.38 ± 2.02a	11.67 ± 1.40a	15.08 ± 1.78b	15.42 ± 3.00b	19.32 ± 2.32c
PL	27.95 ± 3.02bc	20.47 ± 0.58a	21.97 ± 2.19a	23.58 ± 2.18ab	26.92 ± 4.82c	27.34 ± 2.87c
SL	11.17 ± 1.23c	7.41 ± 0.54a	9.02 ± 1.17b	11.56 ± 1.17c	12.09 ± 1.42c	16.92 ± 2.07d
OL	18.89 ± 1.06ab	17.43 ± 2.09a	23.48 ± 3.00b	52.78 ± 7.91e	29.63 ± 5.66c	41.28 ± 9.22d
OD	15.3 ± 0.86ab	14.52 ± 2.01a	15.28 ± 1.31a	19.75 ± 2.40b	22.75 ± 3.63c	34.19 ± 6.33d

Staminate	CF (*N* = 10)	CPF (*N* = 8)	CAS (*N* = 34)	CPP (*N* = 35)	CAA (*N* = 47)	CM (*N* = 32)
CD	53.82 ± 3.55a	55.30 ± 10.84a	74.04 ± 11.34b	85.75 ± 14.15c	99.17 ± 20.82d	95.99 ± 9.93cd
TL	39.46 ± 3.61a	39.34 ± 4.06a	41.85 ± 4.66a	42.31 ± 7.74a	52 ± 8.03b	65.22 ± 7.32c
CL	62.05 ± 5.85ab	56.73 ± 6.57a	71.97 ± 9.66bc	76.33 ± 12.36c	88.84 ± 14.14d	97.28 ± 10.12e
TD1	18.13 ± 1.04bc	12.47 ± 2.27a	14.02 ± 1.86a	18.78 ± 3.28c	18.84 ± 3.45c	16.36 ± 1.60b
TD2	26.19 ± 2.59ab	21.76 ± 3.61a	22.54 ± 2.97a	29.06 ± 4.70b	32.70 ± 4.95c	27.76 ± 4.01b
TD3	34.15 ± 5.35a	36.74 ± 9.05a	41.70 ± 6.90a	47.78 ± 8.93b	60.17 ± 12.92c	61.02 ± 6.81c
NDm	7.47 ± 1.37ab	6.30 ± 1.15a	7.93 ± 1.11b	12.66 ± 2.52d	9.53 ± 1.43c	9.43 ± 1.02c
AD	5.37 ± 0.73bc	4.84 ± 0.33ab	4.54 ± 0.43a	5.78 ± 0.87c	4.97 ± 0.67b	5.37 ± 0.33bc
StL	28.80 ± 3.76b	23.16 ± 2.47a	24.65 ± 2.92a	24.22 ± 3.44a	25.84 ± 3.21ab	33.15 ± 2.41c
AL	20.92 ± 3.72c	10.57 ± 1.43a	13.33 ± 1.93b	10.95 ± 2.35a	13.51 ± 2.30b	21.43 ± 1.91c

### 3.2 Nectar Traits

**FIGURE 3:**
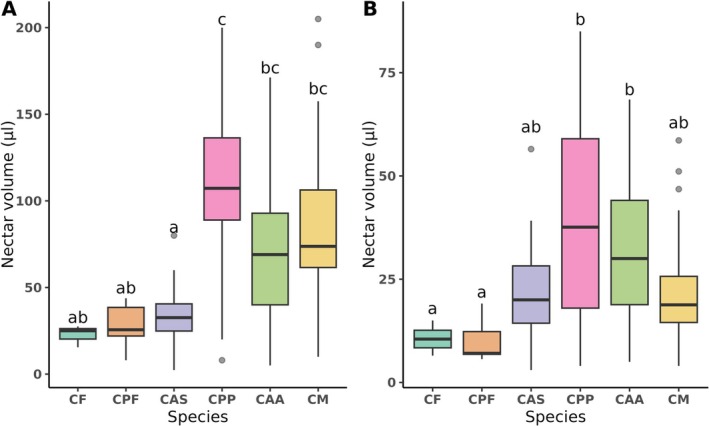
Nectar volume (μL) by Cucurbita species of (A) pistillate and (B) staminate flowers. Different letters indicate differences between species. Abbreviations as in Figure 2.

**FIGURE 4:**
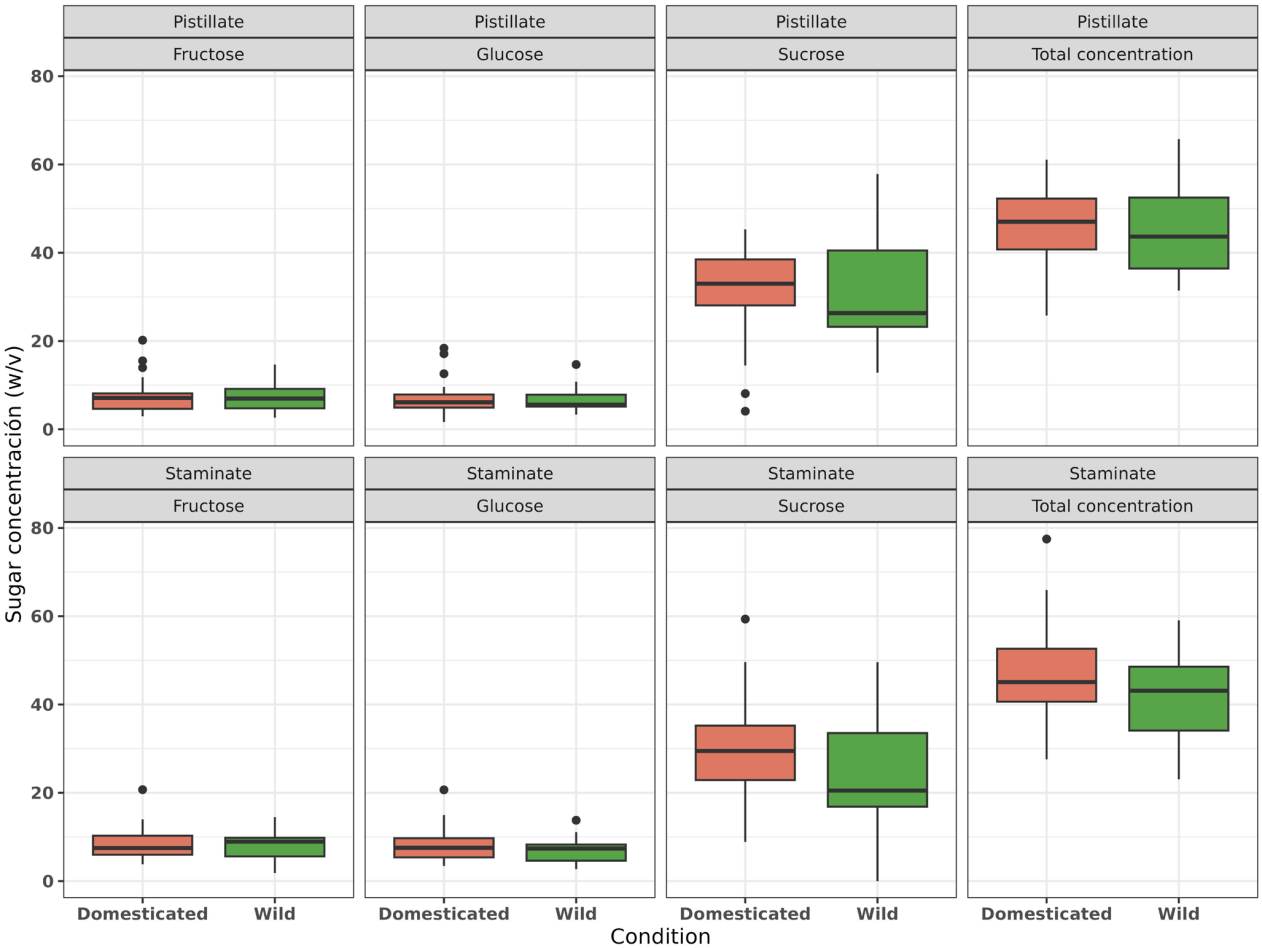
Sugar nectar concentrations (fructose, glucose, sucrose, and total sugar concentration) grouped by conditions (wild and domesticated) and by floral sex.

We observed an increase in nectar volume in both pistillate and staminate flowers in domesticated species compared to wild species (*χ* ^2^ = 73.70, df = 5, *p* < 0.001). Between floral sexes, we observe significant differences in nectar volume (*χ* ^2^ = 82.89, df = 1, *p* < 0.001). In both floral sexes, the domesticated species CPP and CAA produced more nectar; meanwhile, the wild species CF and CPF produced the lowest nectar volume (Figure 3). The nectar sugar composition did not show statistically significant differences between conditions (Figure 4) in total sugar concentration, but when analyzed among species, we observed statistically significant differences (*χ* ^2^ = 34.51, df = 4, *p* < 0.001; Table 2). The wild species CPF had the nectar with the highest total sugar concentration (mean 58.88% ± SD 6.23%), while the wild species CAS had the lowest concentration (40.02% ± 5.82%); the other species had similar total sugar concentrations (Table 2).

**TABLE 2:** Mean (±SD) of nectar volume, total sugar concentration, and each type of sugar (fructose, glucose, and sucrose) of pistillate and staminate flowers of domesticated and wild *Cucurbita* species. Different letters within a column indicate differences between species.

Pistillate		Nectar traits				
Conditions	Species	Volume (μL)	Fructose (w/v)	Glucose (w/v)	Sucrose (w/v)	Total sugar (w/v)
Wild	CF	22.66 ± 6.33 (3)	—	—	—	—
Wild	CPF	27.58 ± 14.14 (5)	4.18 ± 1.11 (4)a	5.02 ± 0.56a	49.68 ± 6.95c	58.88 ± 6.23b
Wild	CAS	32.44 ± 16.35 (27)	8.72 ± 3.20 (10)a	7.41 ± 3.29a	23.89 ± 5.14a	40.02 ± 5.82a
Domesticated	CPP	107.45 ± 48.79 (26)	9.21 ± 6.49 (5)a	7.17 ± 6.45a	23.89 ± 12.59ab	40.28 ± 9.40a
Domesticated	CAA	65.64 ± 38.27 (31)	9.20 ± 3.25 (9)a	8.68 ± 3.75a	29.02 ± 11.00ab	46.91 ± 9.97ab
Domesticated	CM	87.11 ± 54.66 (20)	5.84 ± 2.54 (10)a	5.58 ± 2.16a	37.31 ± 5.96bc	48.73 ± 5.94ab
**Staminate**						
Wild	CF	10.60 ± 2.98 (10)	—	—	—	—
Wild	CPF	10.01 ± 5.27 (8)	3.53 ± 1.63 (5)a	4.07 ± 1.34a	45.44 ± 6.46c	53.05 ± 5.55b
Wild	CAS	21.59 ± 10.86 (35)	9.42 ± 2.00 (12)b	7.78 ± 2.21a	17.64 ± 8.74a	34.85 ± 7.74a
Domesticated	CPP	40.43 ± 24.29 (31)	7.29 ± 2.71 (4)ab	7.17 ± 2.11a	27.73 ± 4.80abc	42.19 ± 0.96ab
Domesticated	CAA	33.59 ± 17.02 (47)	8.73 ± 5.01 (10)b	8.22 ± 5.56a	28.96 ± 11.66ab	45.93 ± 8.90b
Domesticated	CM	21.68 ± 12.86 (32)	8.11 ± 2.96 (12)b	8.45 ± 3.27a	31.85 ± 14.86bc	48.42 ± 14.82b

When we analyzed different sugars, we observed that sucrose was the most abundant nectar sugar in both domesticated and wild species and differed in concentration among species (*χ* ^2^ = 53.80, df = 4, *p* < 0.001) but not between conditions (Figure 4). The wild species CPF had the highest sucrose concentration. Fructose and glucose were four and three orders of magnitude lower than sucrose, respectively (Table 2). The concentrations of fructose (*χ* ^2^ = 27.87, df = 4, *p* < 0.001) and glucose (*χ* ^2^ = 10.69, df = 4, *p* = 0.03) differ significantly among species but not between conditions (Figure 4). The wild species CPF had the lowest concentration of both fructose and glucose; the other species showed similar fructose and glucose concentrations (Table 2). We did not observe statistically significant differences between sexes in concentrations of sucrose (*χ* ^2^ = 2.12, df = 1, *p* = 0.14), fructose (*χ* ^2^ = 0.30, df = 1, *p* = 0.58), or glucose (*χ* ^2^ = 0.77, df = 1, *p* = 0.37).

**FIGURE 5:**
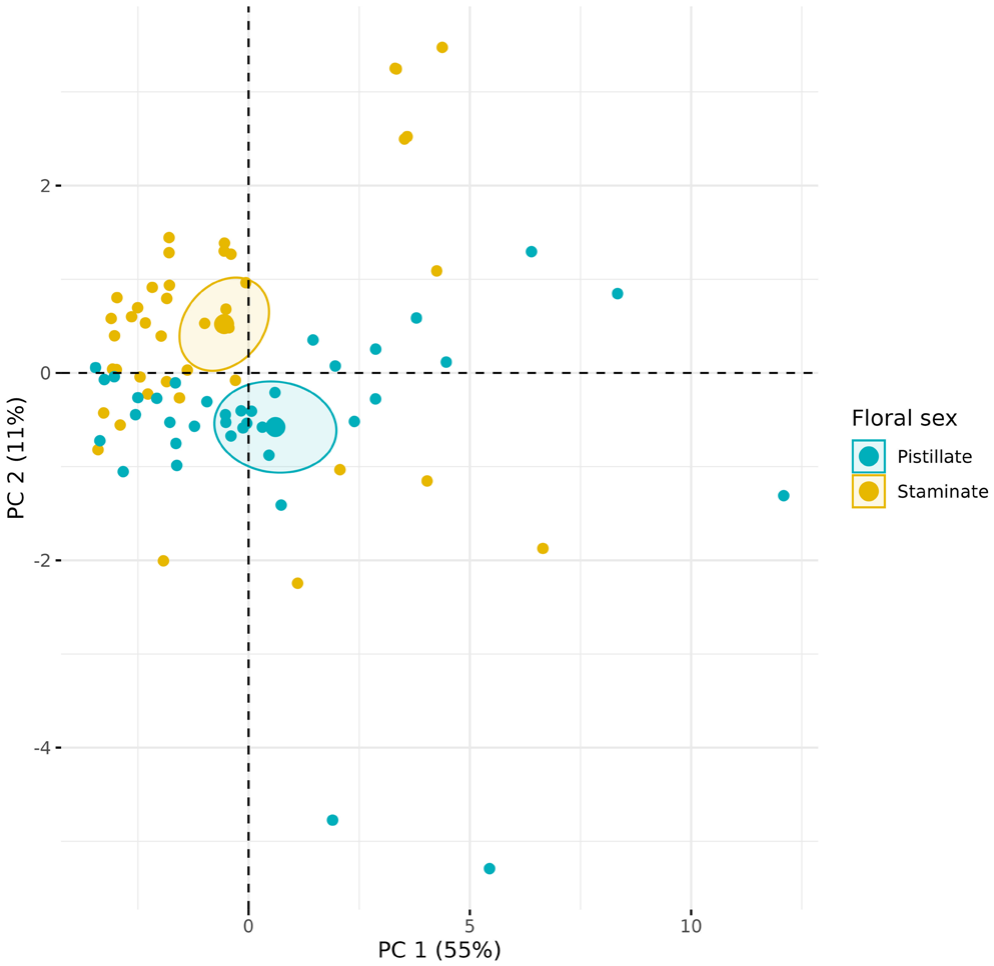
PCA of amino acid concentrations by floral sex, combining all *Cucurbita* species in this study.

We could not secure nectar from pistillate CF flowers; therefore, we excluded this species from this analysis. The estimated 17 amino acids were present in almost all the tested samples, except for threonine, which was not found in pistillate flowers of the species CPF and CPP (Table S1). The PC1 explained 55% of the variance, and the amino acids that contributed the most to this variation were Ile, Tyr, and Leu. The PC2 explained 11% of the variance, and the traits that contributed most to variation were His, Cys, and Asp. Amino acid concentrations did not differ between conditions (*F* = 0.35, df = 1, *p* = 0.79) nor among domesticated and wild species (*F* = 1.11, df = 5, *p* = 0.29) but did differ between floral sexes (*F* = 5.79, df = 1, *p* < 0.001; Figure 5).

### 3.3 Pollen Traits

**FIGURE 6:**
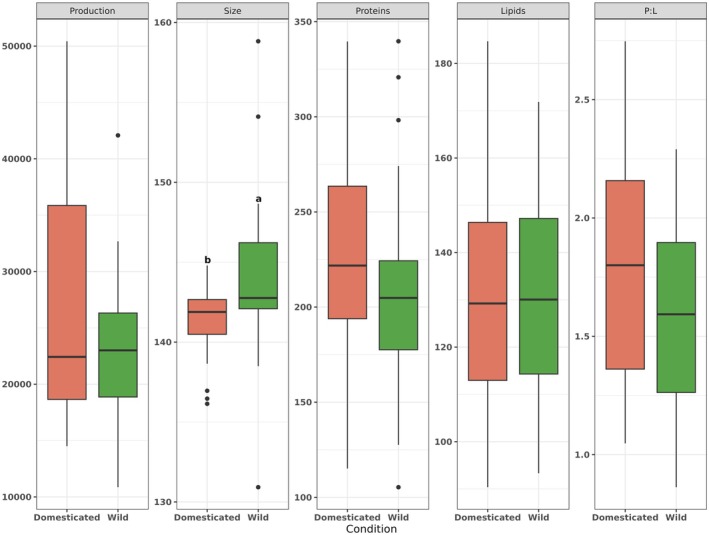
Pollen traits (pollen production and size, protein and lipid concentrations, and the protein: lipid ratio) grouped by conditions: wild and domesticated. Different letters indicate differences between conditions.

*Cucurbita* species had significant differences in pollen production (*χ* ^2^ = 164.83, df = 5, *p* < 0.001, Table 3), with the domesticated species CM having the highest pollen production (40,137 ± 5872), followed by the wild species CF (28,448 ± 6578). On the other hand, the species that produced the lowest quantity of pollen was the wild species CPF (13,408 ± 2967), followed by the domesticated species CPP (16,855 ± 1614). When we analyzed pollen production by conditions, we did not observe statistically significant differences, but we did observe significant differences in pollen size (Figure 6). The analysis of pollen grain size revealed significant differences among species (*χ* ^2^ = 27.04, df = 5, *p* < 0.001), with the pollen grains of the wild species CF being the largest of all species (148.75 ± 1.83 μm). All other species did not differ significantly in pollen grain sizes (Table 3).

**TABLE 3:** Mean (±SD) of pollen traits of wild and domesticated *Cucurbita* species. Different letters within a row indicate differences between species.

Pollen traits	*Cucurbita* species					
	Wild			Domesticated		
	CF	CPF	CAS	CPP	CAA	CM
Pollen production	28,448 ± 6578 (8)c	13,408 ± 2967 (4)a	22,033 ± 3906 (14)bc	16,855 ± 1614 (9)ab	23,457 ± 5485 (13)c	40,137 ± 5872 (11)d
Pollen size (μm)	148.75 ± 5.19 (8)b	137.78 ± 4.71 (4)a	142.93 ± 1.63 (14)a	140.72 ± 1.78 (9)a	141.51 ± 1.87 (13)a	141.69 ± 2.66 (11)a
Pollen protein (μg/mg)	213.45 ± 74.97 (10)	217.83 ± 55.21 (8)	191.13 ± 29.01 (11)	212.96 ± 72.25 (8)	228.46 ± 51.02 (11)	237.05 ± 40.74 (15)
Pollen lipid (μg/mg)	135.66 ± 22.66 (10)ab	139.67 ± 21.14 (8)ab	120.46 ± 17.82 (11)a	116.33 ± 17.88 (8)a	116.26 ± 13.30 (11)a	152.05 ± 19.94 (15)b
P:L	1.60 ± 0.44 (10)	1.56 ± 0.44 (8)	1.63 ± 0.32 (11)	1.74 ± 0.52 (7)	2.00 ± 0.50 (11)	1.62 ± 0.39 (15)

Regarding pollen chemistry, we observed no statistically significant differences between conditions in protein or lipid concentrations or P:L ratios (Figure 6). We neither observed statistically significant differences among species in protein concentration (*χ* ^2^ = 5.14, df = 5, *p* = 0.39) or P:L ratios (*χ* ^2^ = 7.28, df = 5, *p* = 0.20). However, we found significant differences in lipid concentration (*χ* ^2^ = 35.52, df = 5, *p* < 0.001), with the domesticated species CM having the highest lipid concentration (152.05 ± 19.94), followed by the wild species CPF (139.67 ± 21.14) and CF (135.66 ± 22.66). Meanwhile, the domesticated CPP, CAA, and wild CAS species had similar lipid concentrations (Table 3).

## 4 Discussion

The *Cucurbita* genus has a long history of cultivation, with archaeological evidence indicating that squash was domesticated approximately 10,000 years ago (Whitaker and Cutler 1971; Whitaker 1981; Decker 1988; Decker‐Walters 1990). Mexico is recognized as the center of origin and domestication for this genus. Most species within this genus are monoecious, making them an ideal system for studying the effects of artificial selection by humans on floral characteristics. This study builds on previous research conducted in our lab by Glasser et al. (2023) and advances our understanding of the effects of domestication on plant floral morphology and floral rewards and the subsequent impacts on plant–pollinator interactions.

Our results indicate that artificial selection is acting on flowers, as we observed an increase in floral morphological traits in both pistillate and staminate flowers of domesticated species compared to the wild *Cucurbita* species evaluated in this study. The distinctive characteristics defining domestication syndromes are derived primarily from studies on cereals (Alam and Purugganan 2024). Meyer et al. (2012) extensively analyzed domestication traits and observed a considerable variation in syndromes depending on plant life cycles and local conditions of domestication centers; however, flowers are not considered a part of domestication traits. Our results show that flowers are affected by selection exerted during domestication, probably as an indirect consequence of selecting larger fruits and seeds (Lira et al. 2016). Our results are comparable to those previously described by Glasser et al. (2023) for three species of the genus *Cucurbita*, who found that domesticated species *C. moschata* and *C. argyrosperma* subsp. *argyrosperma* had larger floral sizes compared to the wild relative *C. argyrosperma* subsp. *sororia*. We also observed larger floral size in the domesticated species *C. pepo* subsp. *pepo* compared to the wild species *C. pepo* subsp. *fraterna* and *C. foetidissima* , species not included in Glasser et al. (2023) study. Other studies on *Elettaria cardamomum* (Kuriakose et al. 2009) and *Physalis philadelphica* (Solís‐Montero et al. 2021) also found an increase in the size of flowers in domesticated plants. We propose that increased flower size is part of the domestication syndrome in *Cucurbita*.

The effects of domestication in our study species were more pronounced in pistillate flowers compared to staminate flowers. This may be because the selective pressure of domestication is stronger on pistillate flowers, as they are responsible for producing fruits and seeds—traits that are the primary targets of selection in squash. However, in staminate flowers, an increase in most floral morphological traits was also observed in domesticated species with respect to wild species. Artificial selection will likely affect both pistillate and staminate flowers, but in different magnitudes. Although *Cucurbita* flowers are unisexual, pistillate and staminate flowers have comparable ontogenetic pathways because both genders initiate from a hermaphrodite floral meristem with the formation of primordia of sepals, petals, stamens, and carpels (Irish and Nelson 1989; Bai et al. 2004; Martínez and Jamilena 2021). However, in the case of pistillate flowers, the development of a carpel is promoted by ethylene while arresting the development of stamens. Ethylene is an essential hormone in sex determination in cucurbits (Segura et al. 2023, 2024). Therefore, domestication may have a stronger selective effect on female reproductive traits, but male floral traits are also affected to a lesser degree.

Larger flowers in domesticated species may also favor the mutualistic interactions with bees of the *Eucera* genus, commonly known as squash bees, which are the primary and most effective pollinators of *Cucurbita* species in their center of origin (Delgado‐Carrillo et al. 2018). For example, Glasser et al. (2023) observed an increase in the visitation of these bees in the flowers of domesticated *Cucurbita* species. Furthermore, the expansion of the distribution range of the squash bees was also facilitated by the expansion of domesticated populations of squash into new regions (Pope et al. 2023).

As we observed in the floral morphology, domesticated species produced more nectar in both pistillate and staminate flowers than in flowers of wild species. Floral nectar is considered a highly variable trait that can vary widely in response to environmental factors, including temperature, relative humidity, light, and soil moisture (Varga and Soulsbury 2017; Parachnowitsch et al. 2019; Venjakob et al. 2022). However, since our study species were all grown in a common garden, environmental conditions were similar for all species. Therefore, the observed increase in nectar volume among domesticated species may be attributed to an allometric relationship between floral morphological traits and nectaries. As these organs increase in size through artificial selection, they may lead to bigger nectaries with greater nectar production (Kuriakose et al. 2009).

Despite differences in nectar volumes between domesticated and wild species, the total sugar concentration and the concentration of each sugar type (i.e., fructose, glucose, sucrose) were comparable between conditions (Figure 4) as well as among all species (Table 2). Nectar sugar composition may be associated with the phylogenetic relationships among species and their pollinator interactions. For example, nectar chemistry may be similar among phylogenetically related taxa (Nicolson and Thornburg 2007; Nepi et al. 2010). On the other hand, the energetic preferences of pollinators may drive the composition of nectar sugars (Baker and Baker 1983; De La Barrera and Nobel 2004; Nicolson and Thornburg 2007; Bogo et al. 2021). The sugar concentration patterns observed in our study species, whether domesticated or wild, are consistent with energy preferences previously described for squash bees (Baker and Baker 1983; Perret 2001). Among the studied species, only *C. pepo* subsp. *fraterna* exhibited significant differences in sugar concentrations. Previous studies have documented sugar concentration variability within the *C. pepo* group. For instance, the ‘Zuchino’ variety of *C. pepo* displayed an elevated sucrose‐to‐hexose ratio relative to other cultivars of the same species. This change in sugar composition increased visitation by bumblebees ( *Bombus terrestris* L.) (Roldán‐Serrano and Guerra‐Sanz 2005). In contrast, the observed similarity in sugar concentration across most of our study species, genders, and conditions (wild & domesticated) can be attributed to their interactions with pollinators and the close phylogenetic relationships among these *Cucurbita* species.

In this study, we did not observe significant differences in the concentration of nectar amino acids between conditions or species. Amino acids are the most abundant nonsugar metabolites in floral nectar (Roy et al. 2017; Nepi et al. 2018). They can have diverse functions, including being a source of nitrogen for pollinators (Blüthgen and Fiedler 2004), protection against microorganisms (Chevrot et al. 2006; Peay et al. 2012), and moderate insect behavior by stimulating insect chemosensory receptors (Nicolson and Thornburg 2007; Petanidou 2007). The similarity in amino acid concentrations between domesticated and wild *Cucurbita* species, like that of nectar sugar concentration, is probably due to phylogenetically conserved traits that may be subject to stabilizing selection that is likely unaffected by domestication (Petanidou 2005; Nepi et al. 2010).

Although the concentrations of nectar amino acids were similar between wild and domesticated species, they differed between floral sexes (Figure 5). These findings are similar to those observed by Chatt et al. (2018), who found differences in the relative concentrations of specific amino acids between staminate and pistillate flowers of *C. maxima* . The observed differences in amino acid concentrations between floral sexes in this and other studies may reflect differences in nectar presentation (Nepi and Pacini 1993; Nepi et al. 1996) and nectar production in *Cucurbita* genus (Nepi et al. 2001; Nepi and Stpiczyńska 2007; Nepi, Cresti, et al. 2011b). Nectar from pistillate flowers is more exposed to environmental conditions due to anatomic differences between sexes (Nepi et al. 1996; Chatt et al. 2018); for example, pistillate flowers have larger nectaries, which may influence nectar reabsorption, which is then reallocated for seed development (Nepi et al. 2001). Another explanation is that pistillate flowers are more susceptible to microorganism contamination than staminate flowers (Nepi, Bini, et al. 2011a), which may affect amino acid concentrations, as amino acids act as antimicrobial agents to maintain nectar quality and prevent the intrusion of pathogens into the vascular system, also protecting initial fruit development (Chevrot et al. 2006; Sasu et al. 2010).

We found differences in pollen production and size between species, but these differences were not consistent between domesticated and wild species (Figure 6). Several studies have found a trade‐off between pollen production and pollen grain size (Vonhof and Harder 1995; Sarkissian and Harder 2001; Yang and Guo 2004; Ejsmond et al. 2015). Thus, species that produce a large quantity of pollen grains have smaller grains, and vice versa. Pollen traits may also have allometric relationships with other flower parts, such as anther length and width (Severova et al. 2022). This is consistent with our result; for example, *C. moschata* has large flowers and produces the most abundant pollen, while species with small flowers, such as *C. argyrosperma* subsp. *sororia* and *C. pepo* subsp. *fraterna*, produce less pollen. The similar sizes of pollen grains found in most of our study species may also reflect the coevolutionary relationship with squash bees. The only species that differed was *C. foetidissima* , which had the greatest pollen size. Squash bees have specialized structures, such as robust unbranched hairs (Roberts and Vallespir 1978; Thorp 1979), suitable for collecting large pollen grains produced by squash species. Thus, the selective pressure from these specialized pollinators likely limits changes in pollen grain size among squash species. Furthermore, these pollen traits arose from coevolution with squash bees and remained stable throughout cultivation.

For chemical pollen traits, we found no differences in protein concentrations between the domesticated and wild species evaluated in this study. Squash bees are oligolectic, that is, the pollen they use to feed their brood is mainly from the genus *Cucurbita* (Hurd et al. 1971; Delgado‐Carrillo et al. 2018; Glasser et al. 2023). Bees ensure a constant supply of high protein quality by limiting their foraging to a smaller number of closely related host plants (Rasmussen et al. 2020). Squash bees may exert a strong selective pressure on pollen nutritional quality to maintain a constant protein content, as pollen nutritional quality changes could affect the health and growth of bee progeny (Roulston and Cane 2000). Similarly, in another oligolectic bee, *Chelostoma florisomne* (Megachilidae), which forages primarily on *Ranunculus* spp., the digestive system evolved to efficiently remove and absorb the nutrients present in the pollen of this plant genus (Dobson and Peng 1997). Additionally, from a plant's perspective, pollen protein concentration is one of the main nutritional elements for the development and pollen tube growth (Ruedenauer et al. 2019). This is particularly important because all of our studied *Cucurbita* species have similar pistil lengths (20.47 to 27.95 mm). Therefore, we expect the pollen of our study species to be equally provisioned with protein in order to develop pollen tubes that effectively fertilize ovules in styles with similar lengths (Quesada et al. 1991, 1993, 2001; Winsor et al. 2000).

Regarding pollen lipid concentrations, we observed significant variation in this trait across our studied taxa unrelated to domestication. These findings agree with previous studies that have observed significant variations in lipid concentration among different families and genera (Vaudo et al. 2020; Russo et al. 2023; Chau and Rehan 2024). This pollen trait may also vary due to environmental factors like temperature or water stress (Russo et al. 2020; Vaudo et al. 2024). From the plant's perspective, pollen lipids are important in preventing desiccation, pollen attachment to the stigma, and interaction and signaling processes (Pacini and Hesse 2005; Ischebeck 2016). Further studies are needed to determine the most important proximate factors that drive the differences in pollen lipid concentrations in our study species.

When we compared the pollen protein–lipid ratio (P:L) among species, we found no significant differences. We discovered higher protein concentrations than lipid concentrations in all the study species, ranging from 1.5:1 to 2:1. These findings are similar to those previously reported for *C. pepo* (Treanore et al. 2019). Additionally, the P:L ratio consumed by squash bees is similar to those observed for bees such as *Apis mellifera* (Vaudo et al. 2020) in contrast to other hymenopterans such as *Bombus*, who collect pollen on average with a P:L ratio of 4:1 (Vaudo et al. 2018). The protein–lipid ratio appears to be a primary factor driving the foraging behavior of floral visitors (Vaudo et al. 2016, 2018; Mokkapati et al. 2024). In addition, as observed by Brochu et al. (2020), *Cucurbita* pollen may have physical, nutritional, and possibly chemical defenses that are capable of imposing severe physiological costs on bees other than squash bees. For example, squash bees visit staminate flowers early in the morning before other bee species, removing a greater amount of pollen (Delgado‐Carrillo et al. 2018). Thus, squash bees may be better adapted to extract most of the nutrients from *Cucurbita* pollen and metabolize chemical defenses, in contrast to other bee species (Praz et al. 2008; Weiner et al. 2010; Dharampal et al. 2022; Feng et al. 2024; Ruedenauer 2024). Therefore, the similarity in P:L ratios among our study species may be strongly affected by its specialized pollinators (Ruedenauer et al. 2019).

In conclusion, the increase in fruit and seed size within the genus *Cucurbita* during domestication has also increased most floral morphological traits in both pistillate and staminate flowers. However, our results indicate that nectar and pollen traits are not equally influenced by domestication. Since the species in our study have a relatively recent evolutionary history (1.33 to 1.06 Ma), nectar and pollen traits may remain conserved across species. Additionally, squash plants have a close coevolutionary relationship with bees of the *Eucera* genus; thus, the nutrient content in the nectar and pollen of our study species may be more responsive to the nutritional needs of their pollinators than to artificial selection during the domestication process.

## Author Contributions

**Luis Alberto Villanueva‐Espino:** conceptualization (equal), data curation (equal), formal analysis (equal), investigation (equal), methodology (equal), validation (equal), visualization (equal), writing – original draft (equal), writing – review and editing (equal). **Irais Avila‐Eulogio:** conceptualization (equal), data curation (equal), formal analysis (equal), investigation (equal), methodology (equal), writing – review and editing (equal). **M. Hesajim de Santiago‐Hernández:** conceptualization (equal), data curation (equal), formal analysis (equal), investigation (equal), methodology (equal), validation (equal), writing – original draft (equal), writing – review and editing (equal). **César Mateo Flores‐Ortiz:** data curation (equal), formal analysis (equal), methodology (equal), validation (equal), visualization (equal), writing – review and editing (equal). **Rafael Lira Saade:** conceptualization (equal), data curation (equal), formal analysis (equal), investigation (equal), methodology (equal), supervision (lead), validation (equal), visualization (equal), writing – original draft (equal), writing – review and editing (equal). **Adonaji Cortés Pérez:** conceptualization (equal), data curation (equal), formal analysis (equal), investigation (equal), methodology (equal), writing – review and editing (equal). **Eric J. Fuchs:** conceptualization (equal), data curation (equal), formal analysis (equal), investigation (equal), methodology (equal), supervision (lead), validation (equal), visualization (equal), writing – original draft (equal), writing – review and editing (equal). **Mauricio Quesada:** conceptualization (equal), formal analysis (equal), funding acquisition (lead), investigation (equal), methodology (equal), project administration (lead), supervision (lead), validation (equal), writing – original draft (equal), writing – review and editing (equal).

## Conflicts of Interest

The authors declare no conflicts of interest.

## Supporting information


**Appendix S1:** ece372028‐sup‐0001‐AppendixS1.xlsx.

**Appendix S2:** ece372028‐sup‐0002‐AppendixS2.docx.

## Data Availability

The data that support the findings of this study are available in the Supporting Information: Appendices S1 and S2 of this article.
