# Supplementary material for: Impact of Domestication on Floral Traits and Rewards: A Comparison Between Wild and Domesticated Squash (Cucurbita)
Source: Ecol Evol. 2025 Aug 29;15(9):e72028. doi: 10.1002/ece3.72028 (PMC12397498; doi:10.1002/ece3.72028)
Supplement: Supplementary file 2 — Appendix S2: ece372028‐sup‐0002‐AppendixS2.docx. [file ECE3-15-e72028-s002.docx]

Figure S1. Reconstructed phylogeny for *Cucurbita* species used in our study, based on Castellanos-Morales et al., (2018). Asterisks show the domesticated species used in this study.


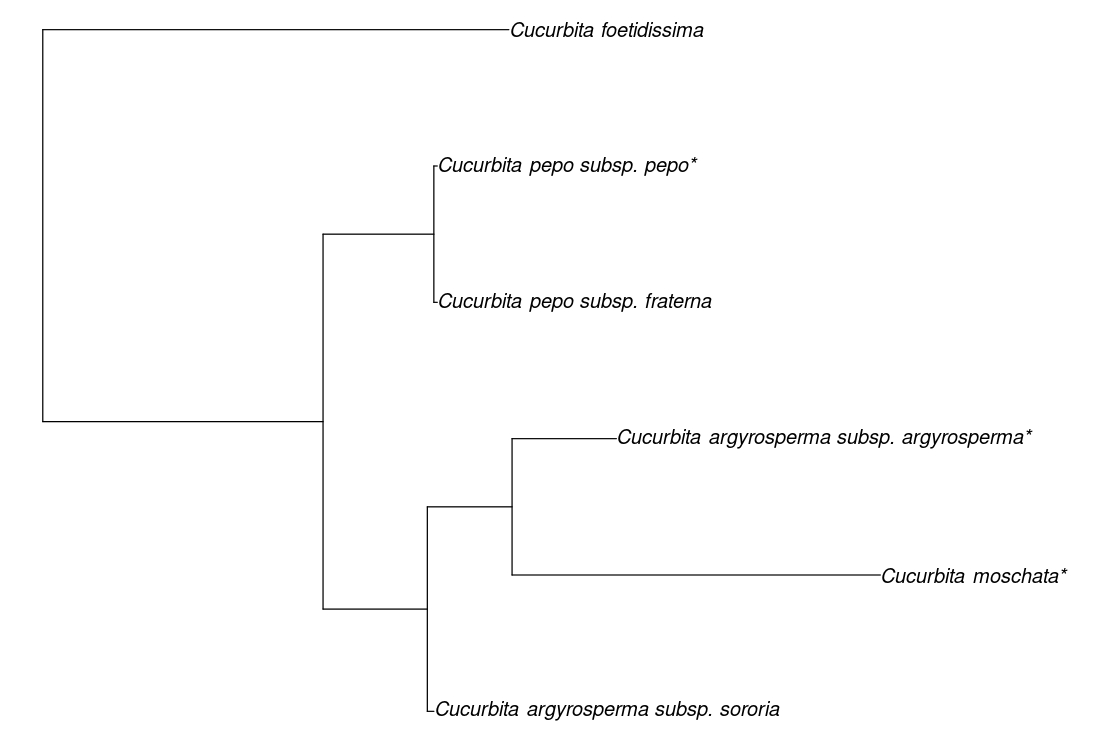


Figure S2. Experimental design used in this study for planting species in the common garden. Abbreviations: *Cucurbita foetidissima* (cf), *Cucurbita pepo* subsp. *fraterna* (cpf), *Cucurbita argyrosperma* subsp. *sororia* (cas), *Cucurbita pepo* subsp. *pepo* (cpp), *Cucurbita argyrosperma* subsp. *argyrosperma* (caa) and *Cucurbita moschata* (cm).


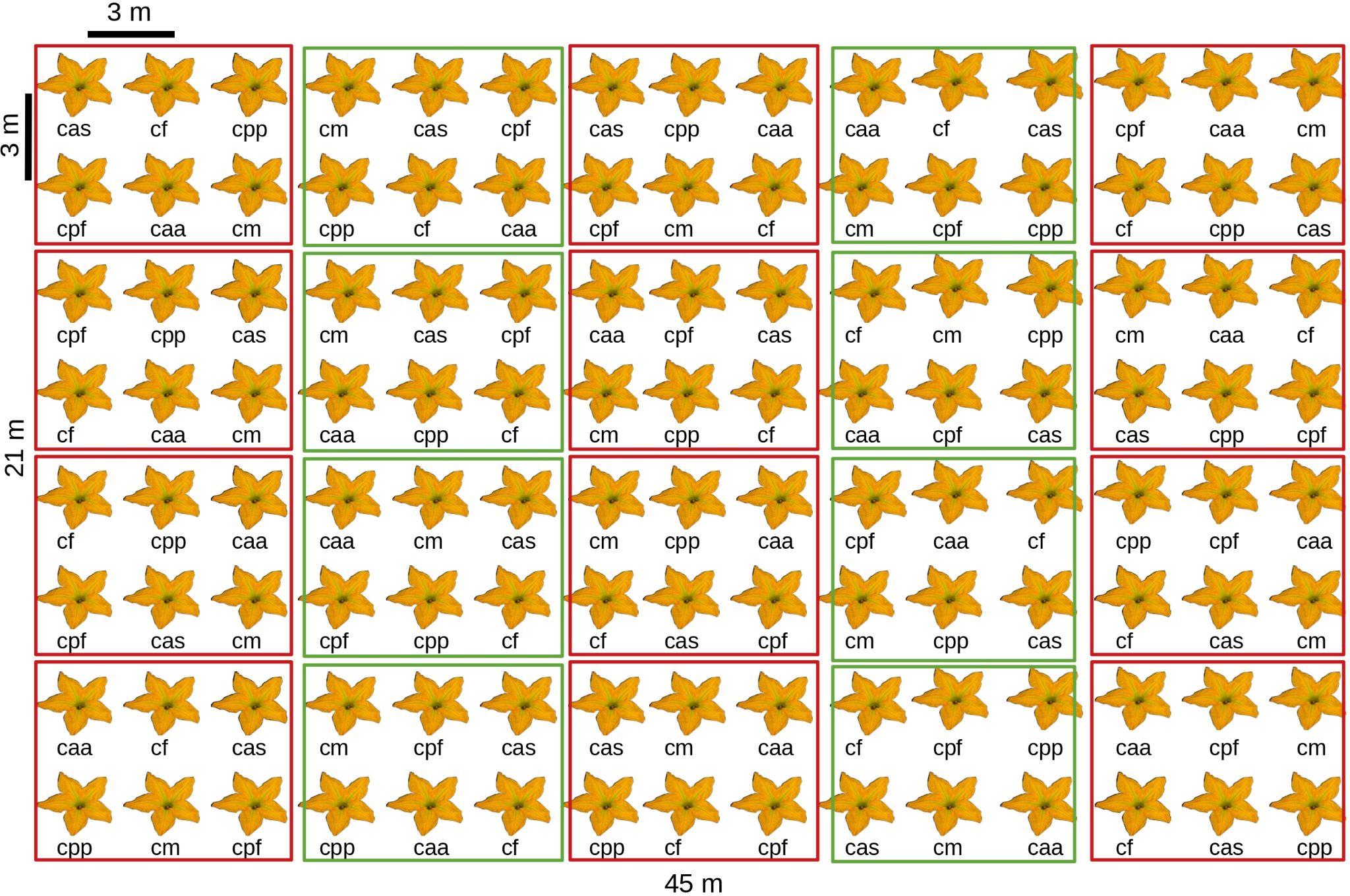


Figure S3. Correlation between the observed total sugar concentration measured with the refractometer and the concentration estimated by the chemometric model.


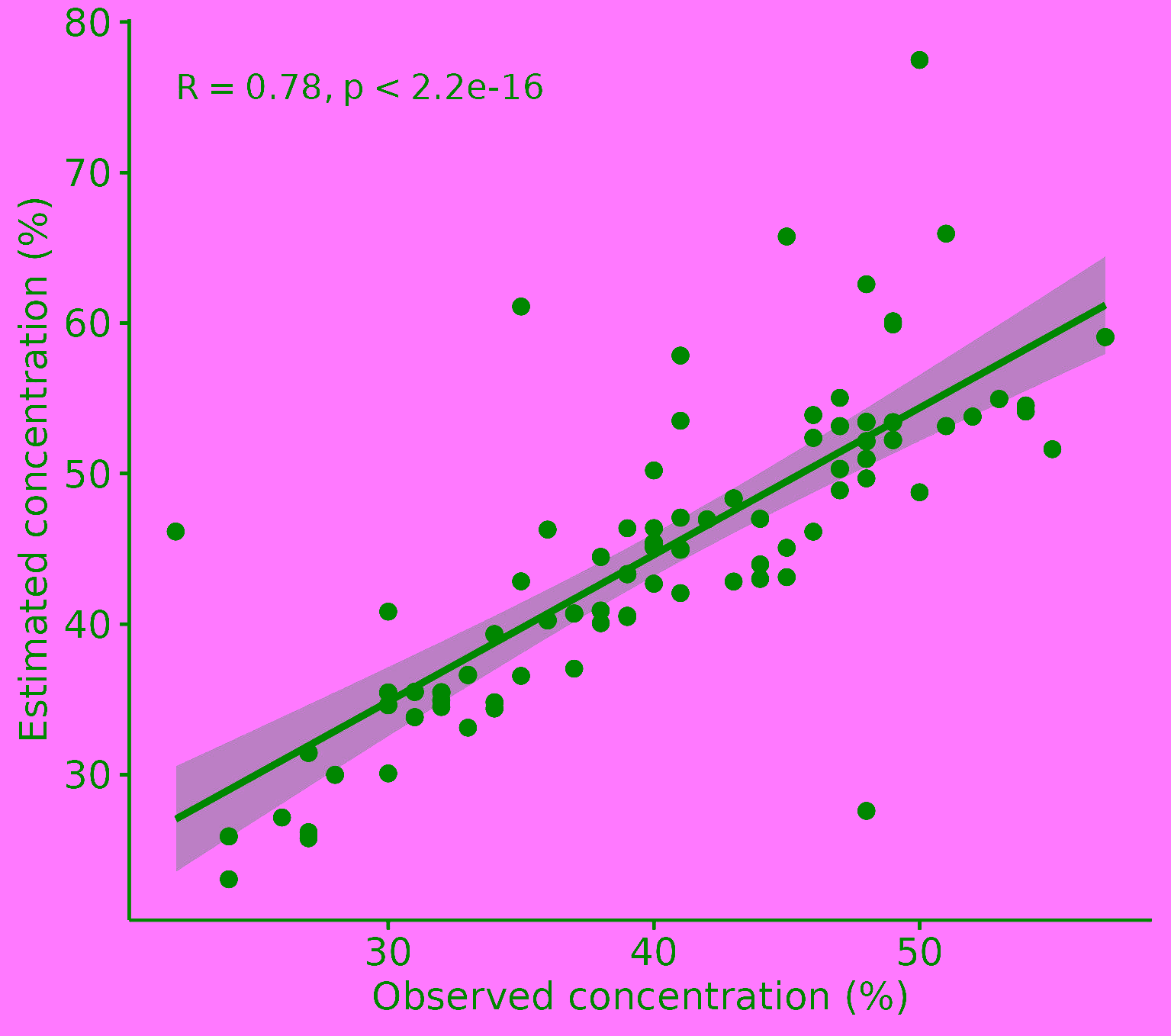


Table S1. Mean ± standard error of amino acid concentrations (μg/ml) for pistillate and staminate *Cucurbita* species analyzed in this study.

| Amino acids | *Cucurbita* species | | | | | | | | | | | |
| --- | --- | --- | --- | --- | --- | --- | --- | --- | --- | --- | --- | --- |
|  | Wild | | | | | | Domesticated | | | | | |
|  | CF | | CPF | | CPP | | CAS | | CAA | | CM | |
|  | **♀** | **♂** | **♀** | **♂** | **♀** | **♂** | **♀** | **♂** | **♀** | **♂** | **♀** | **♂** |
| Asp |  | 7.27±1.34 | 6.22±1.62 | 7.00±3.78 | 31.96±20.99 | 11.88±6.03 | 19.19±7.57 | 7.14±0.72 | 15.18±8.10 | 7.91±1.37 | 12.04±2.04 | 7.45±0.92 |
| Glu |  | 17.86±1.54 | 14.70±4.43 | 27.18±7.04 | 62.99±43.08 | 27.67±3.51 | 44.98±17.40 | 23.60±5.06 | 36.97±13.60 | 28.78±7.54 | 35.42±4.08 | 19.89±2.70 |
| Ser |  | 11.09±1.36 | 4.46±0.98 | 6.13±3.04 | 6.99±2.15 | 6.76±2.12 | 8.41±1.25 | 9.17±2.03 | 8.75±3.87 | 10.03±2.98 | 17.27±10.05 | 5.18±0.71 |
| His |  | 18.54±8.23 | 6.23±0.74 | 2.73±0.75 | 9.05±1.40 | 3.12±0.60 | 3.77±1.06 | 46.63±11.42 | 3.46±1.07 | 43.51±11.99 | 2.93±1.52 | 16.73±5.98 |
| Gly |  | 5.46±2.20 | 3.99±0.55 | 4.38±1.98 | 4.73±0.76 | 3.98±1.11 | 6.73±0.67 | 2.82±0.38 | 5.14±1.52 | 3.29±0.54 | 6.16±0.40 | 2.51±0.30 |
| Thr |  | 1.93±1.93 | 0 | 4.25±1.72 | 0 | 5.48±1.79 | 7.96±0.62 | 4.61±0.79 | 5.81±1.43 | 5.65±1.17 | 7.5±1.15 | 3.49±0.46 |
| Arg |  | 6.99±3.22 | 5.21±0.53 | 6.08±1.83 | 7.69±1.05 | 19.44±5.03 | 9.57±1.12 | 5.35±1.03 | 6.56±2.12 | 13.26±3.47 | 9.11±0.97 | 3.45±0.42 |
| Ala |  | 33.56±2.51 | 12.81±3.10 | 20.64±11.15 | 18.45±6.14 | 5.21±2.17 | 22.66±5.07 | 29.05±6.72 | 25.32±8.98 | 23.80±8.45 | 17.05±4.79 | 21.56±4.24 |
| Tyr |  | 7.76±2.92 | 3.97±1.24 | 2.75±1.32 | 6.49±0.96 | 3.69±1.53 | 8.15±1.24 | 4.59±1.03 | 5.94±2.12 | 5.12±1.19 | 7.90±1.60 | 3.08±0.56 |
| Cys |  | 2.48±1.72 | 1.10±0.44 | 2.61±0.88 | 2.36±0.48 | 4.94±1.16 | 1.96±0.19 | 0.50±0.05 | 3.19±0.41 | 1.52±0.47 | 2.41±0.14 | 1.04±0.27 |
| Val |  | 8.53±0.05 | 3.28±1.24 | 5.92±3.04 | 6.72±1.75 | 8.14±3.26 | 15.89±3.06 | 11.64±2.09 | 11.63±4.25 | 10.95±2.41 | 10.48±2.15 | 6.53±1.39 |
| Met |  | 6.15±4.59 | 3.22±1.07 | 2.17±1.05 | 6.94±0.98 | 2.08±0.61 | 4.59±0.60 | 3.38±1.02 | 3.13±0.99 | 3.66±1.06 | 5.66±0.53 | 2.23±0.35 |
| Phe |  | 11.01±1.18 | 4.13±1.55 | 3.88±0.96 | 6.35±1.32 | 5.26±1.76 | 7.51±109 | 5.49±0.85 | 6.47±1.84 | 4.43±0.67 | 8.41±1.12 | 5.92±1.15 |
| Ile |  | 5.21±1.16 | 3.02±0.96 | 4.35±2.29 | 5.70±1.50 | 6.00±2.59 | 6.92±1.35 | 6.28±1.20 | 6.51±2.10 | 4.32±0.79 | 7.80±1.75 | 3.82±0.85 |
| Leu |  | 9.43±1.49 | 3.79±1.29 | 6.08±2.40 | 6.53±1.76 | 8.70±3.59 | 8.52±1.84 | 10.29±1.69 | 9.41±2.49 | 7.56±1.60 | 8.88±2.23 | 7.97±1.39 |
| Lys |  | 3.48±1.54 | 2.23±0.86 | 3.51±1.89 | 3.77±0.86 | 5.45±2.77 | 3.35±0.62 | 3.13±0.50 | 3.69±0.82 | 3.04±0.52 | 4.15±0.71 | 2.53±0.39 |
| Pro |  | 7.68±0.49 | 2.44±0.71 | 3.67±1.14 | 5.04±1.76 | 5.45±1.94 | 9.51±2.18 | 10.18±2.46 | 8.78±3.40 | 2.98±0.84 | 9.33±2.37 | 7.11±1.49 |

Table S2.- Phylogenetic signal of floral traits from pistillate flowers.

| Traits | Pagel’s 𝝀 | p-value | Blomberg’s K | p-value |
| --- | --- | --- | --- | --- |
| CD | 7.33E-05 | 1.00 | 0.07 | 0.51 |
| TL | 7.33E-05 | 1.00 | 0.59 | 0.14 |
| CL | 7.33E-05 | 1.00 | 0.10 | 0.42 |
| TD1 | 7.33E-05 | 1.00 | 0.03 | 0.75 |
| TD2 | 7.33E-05 | 1.00 | 0.04 | 0.65 |
| TD3 | 7.33E-05 | 1.00 | 0.09 | 0.46 |
| NDf | 7.33E-05 | 1.00 | 0.03 | 0.69 |
| SD | 7.33E-05 | 1.00 | 0.06 | 0.51 |
| PL | 7.33E-05 | 1.00 | 0.08 | 0.45 |
| SL | 7.33E-05 | 1.00 | 0.06 | 0.51 |
| OL | 7.33E-05 | 1.00 | 0.02 | 0.89 |
| OD | 7.33E-05 | 1.00 | 0.22 | 0.32 |
| VN | 7.33E-05 | 1.00 | 0.02 | 0.81 |
| fructose | 3.89E-01 | 0.71 | 0.06 | 0.59 |
| glucose | 8.05E-01 | 0.18 | 0.22 | 0.33 |
| sucrose | 7.33E-05 | 1.00 | 0.05 | 0.54 |
| total sugar | 7.28E-01 | 0.21 | 0.15 | 0.53 |
| Asp | 7.33E-05 | 1.00 | 0.02 | 0.88 |
| Glu | 7.33E-05 | 1.00 | 0.02 | 0.87 |
| Ser | 8.94E-01 | 0.18 | 0.52 | 0.07 |
| His | 7.30E-01 | 0.46 | 0.11 | 0.30 |
| Gly | 9.46E-01 | 0.10 | 0.65 | 0.08 |
| Thr | 1.00E+00 | 0.11 | 0.71 | 0.09 |
| Arg | 7.20E-01 | 0.29 | 0.20 | 0.35 |
| Ala | 8.35E-01 | 0.18 | 0.26 | 0.19 |
| Tyr | 6.75E-01 | 0.33 | 0.15 | 0.40 |
| Cys | 5.26E-01 | 0.53 | 0.11 | 0.43 |
| Val | 7.33E-05 | 1.00 | 0.20 | 0.16 |
| Met | 7.33E-05 | 1.00 | 0.05 | 0.68 |
| Phe | 7.75E-01 | 0.19 | 0.21 | 0.29 |
| Ile | 6.98E-01 | 0.29 | 0.14 | 0.40 |
| Leu | 7.89E-01 | 0.20 | 0.21 | 0.26 |
| Lys | 6.88E-01 | 0.24 | 0.13 | 0.49 |
| Pro | 7.99E-01 | 0.32 | 0.23 | 0.19 |

Table S3.- Phylogenetic signal of floral traits from staminate flowers.

| Traits | Pagel’s 𝝀 | p-value | Blomberg’s K | p-value |
| --- | --- | --- | --- | --- |
| CD | 7.33E-05 | 1.00 | 0.05 | 0.56 |
| TL | 9.56E-01 | 0.49 | 0.73 | 0.14 |
| CL | 7.33E-05 | 1.00 | 0.08 | 0.50 |
| TD1 | 7.33E-05 | 1.00 | 0.02 | 0.98 |
| TD2 | 7.33E-05 | 1.00 | 0.03 | 0.82 |
| TD3 | 7.33E-05 | 1.00 | 0.14 | 0.31 |
| NDm | 7.33E-05 | 1.00 | 0.01 | 0.94 |
| AD | 7.33E-05 | 1.00 | 0.02 | 0.93 |
| StL | 9.70E-01 | 0.38 | 0.53 | 0.07 |
| AL | 1.00E+00 | 0.03 | 1.04 | 0.01 |
| VN | 7.33E-05 | 1.00 | 0.02 | 0.89 |
| fructose | 7.33E-05 | 1.00 | 0.05 | 0.57 |
| glucose | 7.33E-05 | 1.00 | 0.04 | 0.63 |
| sucrose | 7.33E-05 | 1.00 | 0.03 | 0.82 |
| total sugar | 7.33E-05 | 1.00 | 0.03 | 0.81 |
| Asp | 7.33E-05 | 1.00 | 0.01 | 0.94 |
| Glu | 1.00E+00 | 0.13 | 0.81 | 0.01 |
| Ser | 9.82E-01 | 0.36 | 0.62 | 0.02 |
| His | 1.00E+00 | 0.29 | 0.46 | 0.15 |
| Gly | 9.24E-01 | 0.14 | 0.55 | 0.04 |
| Thr | 6.49E-01 | 0.43 | 0.13 | 0.36 |
| Arg | 7.33E-05 | 1.00 | 0.02 | 0.88 |
| Ala | 7.33E-05 | 1.00 | 0.04 | 0.65 |
| Tyr | 8.91E-01 | 0.14 | 0.38 | 0.12 |
| Cys | 7.33E-05 | 1.00 | 0.04 | 0.76 |
| Val | 7.33E-05 | 1.00 | 0.08 | 0.43 |
| Met | 1.00E+00 | 0.01 | 1.66 | 0.01 |
| Phe | 8.81E-01 | 0.08 | 0.37 | 0.27 |
| Ile | 7.33E-05 | 1.00 | 0.04 | 0.63 |
| Leu | 7.33E-05 | 1.00 | 0.03 | 0.74 |
| Lys | 7.33E-05 | 1.00 | 0.02 | 0.71 |
| Pro | 7.33E-05 | 1.00 | 0.14 | 0.24 |
| production | 9.65E-01 | 0.33 | 0.50 | 0.06 |
| size | 7.53E-01 | 0.26 | 0.18 | 0.35 |
| protein | 7.33E-05 | 1.00 | 0.03 | 0.78 |
| lipid | 7.33E-05 | 1.00 | 0.04 | 0.67 |
| PL | 7.33E-05 | 1.00 | 0.26 | 0.42 |
